# Lipophilic Toxin Profile in *Mytilus galloprovincialis* during Episodes of Diarrhetic Shellfish Poisoning (DSP) in the N.E. Adriatic Sea in 2006 

**DOI:** 10.3390/molecules16010888

**Published:** 2011-01-21

**Authors:** Zivana Nincevic Gladan, Ivana Ujevic, Anna Milandri, Ivona Marasovic, Alfiero Ceredi, Silvia Pigozzi, Jasna Arapov, Sanda Skejic

**Affiliations:** 1 Institute of Oceanography and Fisheries, Šet. I. Meštrovića 63, 21000 Split, Croatia; E-Mails: ujevic@izor.hr (I.U.); marasovic@izor.hr (I.M.); arapov@izor.hr (J.A.); sanda@izor.hr (S.S.); 2 Fondazione Centro Ricerche Marine National Reference Laboratory on Marine Biotoxins, 47042 Cesenatico, Italy; E-Mails: anna.milandri@centroricerchemarine.it (A.M.); alfiero.ceredi@centroricerchemarine.it (A.C.); silvia.pigozzi@centroricerchemarine.it (S.P.)

**Keywords:** diarrhetic shellfish poisoning, okadaic acid, *Dinophysis fortii*, Adriatic sea

## Abstract

*Dinophysis* spp. blooms and related shellfish toxicity events of diarrhetic shellfish poisoning (DSP) have been the most reported toxicity event through the Croatian National monitoring program. With the aim to characterize the DSP toxin profile in shellfish farmed in Croatia, for the first time a complete analysis of the toxin profile of Croatian mussels has been carried out using the LC-MS/MS technique. The obtained results showed okadaic acid (OA) as the main toxin contaminating Croatian mussels at that time. The maximum concentration of OA in shellfish tissue was recorded 12 days after the *Dinophysis fortii* bloom, thus suggesting that rapid growth of the toxin level in the shellfish occurred in the first week after the bloom while it was slower in the second week. Furthermore, the presence of only OA at concentrations which could endanger human health suggests *D*. *fortii* as the main organism responsible for the toxic event that occurred in Lim Bay. The presence of gymnodimine and spirolides in Croatian mussel has been detected for the first time, while the presence of yessotoxin and pectenotoxin-2 is confirmed.

## 1. Introduction

*Dinophysis* spp. blooms and related shellfish toxicity events of diarrhetic shellfish poisoning (DSP) are reported worldwide due to their impact on aquaculture and human health. DSP toxins are heat-stable polyether, lipophilic compounds isolated from various species of shellfish and dinoflagellates [1]. The main vector for human intoxication by phycotoxins is consumption of shellfish. The different chemical types of toxins, which are associated with the DSP syndrome, were initially classified into three groups: acidic toxins, including okadaic acid (OA) and its derivatives named dinophysistoxins (DTXs); neutral toxins consisting of polyether-lactones of the pectenotoxin group (PTXs) and sulphated compounds called yessotoxin and its derivates (YTXs). YTXs have now been categorized separately because they do not induce diarrhea [2], while a new group of toxins, called azaspiracids (AZAs), and has had to be added. Although the presence of OAs, PTXs and YTXs have been well documented in the Adriatic Sea [1,3,4,5,6,7] most of the studies concern the western Adriatic coast. Gymnodimines (GYMs) and spirolides are emerging lipophilic marine toxins that belong to a heterogeneous group of macrocyclic compounds called cyclic imines [8]. Since their discovery in the early 1990s, gymnodimines and spirolides have been demonstrated to have a global distribution including Adriatic Sea [9]. These toxins are well known due its “fast acting toxicity” in mouse bioassay.

The eastern part of the Adriatic Sea has been very poorly characterised so far, where research in the field of shellfish toxicity has been carried out mostly in the central area. DSP toxicity has been confirmed [10] although toxin profile data were barely detected, showing low concentrations of OA, DTX-1 and DTX-2 [11,12], which could not explain the toxicity events. In Croatian waters only PTXs were found at concentrations which could be associated with mouse bioassay positive results [12,13]. After the establishment of a national monitoring program in 2004, regular analysis of shellfish revealed that DSP toxicity events appear more often in the northern Adriatic (late summer and autumn), while their appearance in the southern part is rare [14]. With the aim of characterising the DSP toxin profile in shellfish farmed in Croatia, we analyzed positive mouse bioassay samples by LC-MS/MS.

## 2. Results and Discussion

**Figure 1:**
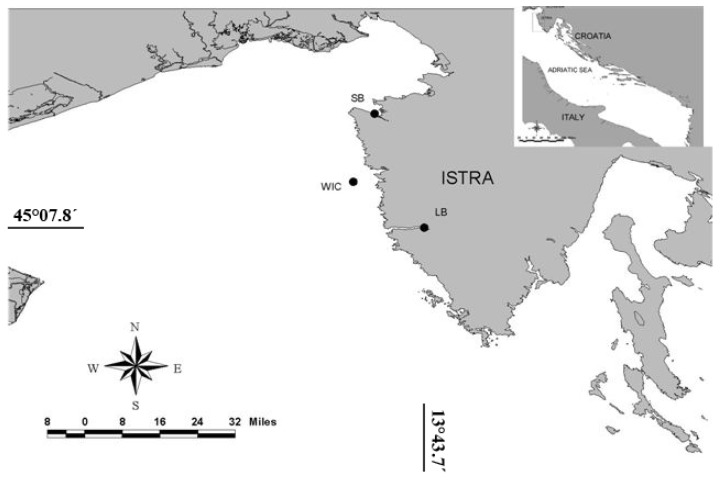
Investigated area with sampling stations.

The national monitoring program of shellfish breading areas revealed DSP toxic episodes in the northern Adriatic during the late summer and autumn period. DSP positive mouse bioassay tests were recorded at three stations: Savudrija basin (SB) and stations located at the western Istrian coast (WIC) and Lim Bay (LB) (Figure 1, Table 1). An exceptionally low survival time was recorded at the WIC station. Throughout the toxic episode only OA was found at a concentration that could be assigned to a positive mouse bioassay (Table 2). Besides toxins cited in table, YTX, PTX-2, putative 7-epi-PTX-2-SA, SPX-1 and GYM were analyzed but the levels found were <LOD or <LOQ. Azaspiracids (AZP), lipophilic toxins which can give positive DSP Mouse Bioassay were not analyzed in this study. Since, dinoflagellate *Protoperidinium crassipes*, and *Azadinium spinosum* which are identified as AZP source, have not been observed in water column at this station, we assumed that investigated shellfish were not contaminated with AZP. 

**Table 1:** Mortality and survival times within 24 h in DSP mouse bioassay.

Station	Date	Mortality	Survival times (h)
SB	09 October	3/3	3.00, 3.00, 4.17
SB	16 October	3/3	4.00, 4.00, 5.00
SB	23 October	2/3	3.00, 3.00
WIC	23 August	3/3	0.02, 0.04, 0.15
LB	29 September	3/3	3.00, 3.25, 3.33
LB	05 October	3/3	1.17, 1.17, 1.25
LB	10 October	3/3	2.00, 2.00, 3.00
LB	17 October	2/3	4.00, 4.17

**Table 2:** Lipophilic toxins levels determined in DSP positive mussel samples.

Station	Date	Free OA (µg/kg)	OA esters (µg/kg)	µg total OA eq./kg	OA esters (%)	homoYTX(mg/kg)	PTX-2-SA (μg/kg)
SB	09/10/06	58	304	362	84	<LOQ	<LOQ
SB	16/10/06	71	75	146	51	<LOQ	<LOQ
SB	23/10/06	94	85	179	48	0.042	<LOQ
WIC	23/08/06	<LOQ	-	78	-	0.130	15
LB	29/09/06	76	237	313	76	<LOD	10
LB	05/10/06	418	669	1087	62	<LOQ	81
LB	10/10/06	307	915	1222	74	<LOQ	<LOQ
LB	17/10/06	79	10	89	11	<LOD	<LOQ

The highest concentration of OA was recorded at the station in Lim Bay and coincided with the shortest mouse survival time when the WIC station was excluded. The Spearman`s Rank correlation between OA concentrations and average mouse survival times (N = 7, r = −0.79, p < 0.05) confirmed OA as the main toxin responsible for shellfish toxicity.

**Figure 2:**
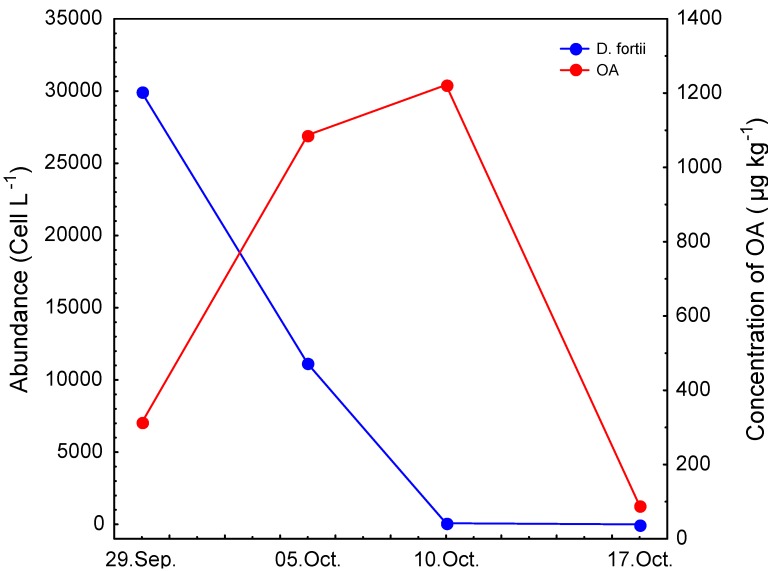
Abundances of *D. fortii* in water samples and concentrations of okadaic acid (OA) in shellfish samples obtained from Lim Bay.

An intensive bloom (1.1–3.0 × 10^4^ cell L^−1^) of *Dinophysis fortii* occurred in Lim Bay at the end of September and beginning of October (Figure 2). At the beginning of the bloom the toxin concentration in the mussel exceeded regulatory toxin limits and maximum concentrations were reached after two weeks. In the first week toxin level growth was rapid and increased 3.5 times (Figure 2). In the second week after the bloom, the toxin level rise was much slower. Seven days after the *D. fortii* bloom terminated, mussel toxin levels fell to 7%.

Bloom of *D. fortii* accompanied by shellfish toxicity recurred in Lim Bay in the autumn of years 2007, 2008 and 2010 (unpublished data). The relation of *D. fortii* to OA shellfish contamination had been previously reported [1,15]. Among *Dinophysis* species, *D. acuminata* is the most studied [16,17,18] and the most reported species that produces OA [19,20,21,22,23], which is probably due to its more intensive and common occurrence. 

Comparison of OA concentrations in mussels determined in this study with these from Thermaikos Gulf during *D. acuminata* bloom, which occurs at a similar intensity [22] as *D. fortii* in Lim Bay, revealed two and three times higher OA concentrations in mussels from Thermaikos Bay than those mussels analyzed in this study. These differences can result from species specific toxin profile, different cellular toxin content or/and the ratio of toxigenic cells to the total particulate matter filtred by the shellfish. Cellular toxin content is variable and depends on many physiological and environmental factors as growth phase, physiological stress (sharp changes in salinity or nutrient deficiency), cellular division, assimilation of amino acids [24,25]. Several authors have postulated that the DSP toxins in *Dinophysis* cells would not be due to synthesis but rather to the ingestion of picophytoplankton which produces the toxins [26,27]. This might explain the great differences in toxin content per cell in *Dinophysis.* Mixotrophic feeding behavior of *Dinophysis* spp. has been pointed as possible explanation of the high variability of toxin content per cell. Relationship between mussel toxin levels and ratio of toxigenic cells to the total particulate matter filtered by the shellfish are based on quantity and quality of food available to the bivalves. In waters low in particulate organic matter, filter feeders need to filter larger volumes to accomplish its nutritional needs. Dahl and Johannessen [28] recommended the use of the ratio between *Dinophysis acuta* and the chlorophyll content as a better index to predict DSP events associated with this species.

The maximum concentration of OA in shellfish tissue was recorded 12 days after the *D. fortii* bloom, thus suggesting that rapid growth of the toxin level in the shellfish occurred in the first week after the bloom while it was slower in the second week. Morano *et al.* [19] recorded a similar result in the Ria de Vigo, Spain where the maximum concentration in shellfish was recorded 16 days after a *D. acuminata* bloom. Seven days after maximal concentrations in shellfish were achieved; levels fell to 7%. This significant loss of OA from shellfish have been accompanied by decrease of toxic cell in water column and appears 7 days after *D. fortii* abundances fall down from 1,1 × 10^4^ to 80 cells L^−1^.

Different depuration rate has been reported in various geographic areas. Svensson [29] reported a depuration rate with an average of 50% reduction after 32 days, which she attributed to the low temperature since the experiment was performed in the Swedish coastal water in November. Slow depuration rate with a semidepuration time of 35–45 days for toxin elimination has been reported by Duinker *et al.* [30] for mussels from Norway. Depuration rate of 50% reduction after 1.5 months was observed Lindahl and Hageltorn [31] in Swedish waters. Faster depuration rates are observed in the southern areas in comparison to Nordic contries. In Spanish waters, Fernandez *et al.* [32] observed 50% reduction in 11–12 days, Blanco *et al.* [33] and Morano *et al.* [19] reported 50% reduction in 7–8 days. In French waters, Marcaillou-Le Baut *et al.* [34] reported 50% reduction of DST in 12 days. Poletti *et al.* [35] recorded semi-depuration time of 3.3 days in Adriatic Sea during sea water temperature above 20 °C. These differences in depuration rates between Nordic and Mediterranean countries could be attributed to temperature diversity, but at least partly it could be a result of different mussels species used. It has been assumed that depuration is faster in higher temperatures due to a generaly higher metabolic activity [36], however, the degree to which temperature affects the uptake and release of toxins is still unknown. The attempts made to obtain precise estimates of temperature effects did not result in any clear conclusion [29,31,33,37] on what could be attributed to the stress effects caused by abrupt temperature changes. The consistent differences in depuration rates have been seen between different seasons [38], with faster depuration rates in summer compared to autumn, an observation that could be associated with temperature influence. Studies on the effect of food quality and availability on depuration rate provide conflicting results. Some studies suggest that the quantity or quality of the food sources affect the depuration rates [34,33], while other studies in field and laboratory suggest that neither food levels, salinity or temperature affect the depuration rates [29,37].

The relationship between toxic phytoplankton and toxin occurrence was not recorded at stations SB and WIC and is probably a consequence of the phytoplankton sampling method. At station SB *Dinophysis rotundata* (80 cells L^−1^) were recorded in September. At station WIC in July *D. sacculus* and *Protoperidinium crassipes* were recorded in abundance of 80 cells L^−1^. In the present study seawater was sampled with a sampling tube where it is possible that big cells were avoided, especially if they were not present in high concentrations unlike at the LB station. Net sampling is more adequate for the detection of low abundance species in seawater for toxic phytoplankton controls [14,39].

Besides OA, low levels (0.13 mg kg ^−1^) of YTX analogs and PTX-2 in some samples were detected (Table 2). The presence of YTX, previously has been reported [40,41]. Although YTX shows high lethality when it is intraperitoneally injected in mice [42] its concentration recorded at the WIC station was too low, in relation to the rapidity of mouse death (Table 1), for it to be attributed to the positive mouse bioassay. These rapid deaths of mice usually were assigned to spirolides and gymnodimine, which were not detected in this sample. It is possible that there were false positive results which may have occurred if the free fatty acids were present [43,44] or acetone and diethyl ether remained in the shellfish extract [45].

Gymnodimine and spirolides, which are known as “fast-acting toxins”, were recorded in Croatian mussels for the first time, but at concentrations below the limit of quantification (<12 µg/kg). Gymnodimine was detected at station SB, while SPX-1 was detected at station LB. Spirolides found at the LB station could be assigned to the presence of *Alexandrium ostenfeldii*, which is the causative organism of spirolide toxins [46,47]. *A. ostenfeldii* was recorded at the LB station seven days prior to the day when spirolide in mussels was detected at concentrations of 6.7 × 10^3^ cell L^−1^. Its concentration fall to 400 cell L^−1^ seven days after spirolides were detected. Gymnodimine presence at the LB station could be related to high abundance of *Gymnodinium* spp. (7.1 × 10^5^ cells L^−1^), which had occured twelve days previously. A wide distribution of Gymnodimine has been reported around the New Zealand coastline [48] and has recently been identified in shellfish from Tunisia [49].

The present study confirmed the continuing presence of PTX in mussels from the Adriatic Sea. Pavela-Vrančić *et al.* [12,50] reported for the first time the presence of the PTX derivative, 7-epi-pectenotoxin-2-seco acid in mussels from the middle Adriatic. This study gives the first evidence of PTX presence in the northern Adriatic and its existence could be related to an intensive bloom of *D. fortii* which is known as the causative organism of PTX [1]. The concentration of PTX was very low (Table 2) indicating that *D. fortii* cells mainly were responsible for OA occurrence in mussels in this instance.

## 3. Experimental

### 3.1. Sampling activities

Mussel (*Mytilus galloprovincialis*) and seawater samples were collected weekly from August to October 2006 at shellfish farms located in the northeastern (NE) Adriatic (Figure 1). Each mussel sample was split in two sub-samples for mouse bioassay (MBA) and LC-MS/MS analysis. Hepatopancreas was used as the test portion for MBA, while the whole flesh tissue was used for the chemical analysis of lipophilic toxins. Integrated seawater samples were collected using a PVC tube sampler for phytoplankton analysis.

### 3.2. Mouse bioassay

Analysis of DSP toxicity by mouse bioassay was performed following the method developed by Yasumoto and collaborators [51]. The method included extraction of 20 g mussel hepatopancreas with acetone. The extract was then evaporated and the residue was partitioned between diethyl ether and water. The organic fraction was evaporated to dryness and the final residue was dissolved in 4 mL 1% Tween 60. Aliquots (1 mL) of this solution were intraperitoneally injected into three mice (18–20 g). Observation time was 24 hours but the test was regarded as positive if at least two out of three mice died within five hours [52,53]. 

### 3.3. Liquid chromatography-mass spectrometry

Further investigations on those samples that gave positive results by the MBA for lipophilic toxins were carried out by liquid chromatography tandem mass spectrometry. Homogenised mussel tissues were extracted once with methanol 90% (sample to solvent ratio = 10). Extracts were then washed with n-hexane and filtered before injection into LC. LC-MS/MS analyses were performed using a 1,200 L triple quadrupole mass spectrometer equipped with an electrospray ionisation source (Varian Inc., Walnut Creek, CA, USA).

Methanol of HPLC grade was purchased from VWR (Milan, Italy). Water was distilled and passed through a MilliQ water purification system (Millipore Ltd., Bedford, MA, USA). Formic acid (reagent grade ≥ 95%) was purchased from Sigma-Aldrich (Steinheim, Germany), while ammonium hydrate for analysis was purchased from Carlo Erba (Milan, Italy). Certified reference materials for OA, DTX-1, PTX-2, YTX, GYM and SPX-1 were purchased from CNRC (Halifax, NS, Canada) and were used for correct identification and quantification of toxins in mussel samples. Analogues of the previously mentioned toxins were identified by direct comparison with contaminated samples of known composition.

Chromatographic separation was performed using a 5 µm SunFire C18, 150 × 2.1 mm column (Waters Corporation, Milford, MA, USA) kept at 30 °C. Mobile phase A consisted of methanol-water (13:87, v/v) containing 50 mM formic acid and 4 mM ammonium hydrate. Mobile phase B consisted of methanol-water (90:10, v/v) containing containing 4.5 mM formic acid and 5.4 mM ammonium hydrate. Flow rate was 200 μL min^−1^. A step gradient elution was used: 0% B for 3 min, 0–97% B in 4 min, 97% B for 15 min. Re-equilibration time at the initial conditions was 8 min. Multiple reaction monitoring (MRM) experiments were carried out in positive/negative switching ion mode in order to investigate the presence of the following toxins: GYM (*m/z* 508 > 490 ES+); SPX-1 (*m/z* 692 > 444; *m/z* 692 > 164 ES+); OA and DTX-2 (*m/z* 803 > 255 ES-); DTX-1 (*m/z* 817 > 255 ES-); PTX-2 (*m/z* 876 > 823 ES+); PTX-1 (*m/z* 892 > 839 ES+); PTX-2-SA and putative 7-epi-PTX-2-SA (*m/z* 894 > 823 ES+); PTX-6 (*m/z* 906 > 853 ES+); YTX (*m/z* 1,141 > 1,061 ES-); homoYTX (*m/z* 1,155 > 1,075 ES-); 45-OHYTX (*m/z* 1,157 > 1,077 ES-); 45-OHhomoYTX (*m/z* 1,171 > 1,091 ES-).

Detection limits were calculated based on a signal/noise (S/N) ratio of 3. LODs: OA 7 µg/kg; DTX-1 7 µg/kg; DTX-2 7 µg/kg; YTX 0.010 mg/kg; homoYTX 0.010 mg/kg; 45-OH YTX 0.010 mg/kg; 45-OH homoYTX 0.010 mg/kg; PTX-2 4 µg/kg; PTX-2-SA 4 µg/kg; putative 7-epi-PTX-2-SA 4 µg/kg; SPX-1 4 µg/kg; GYM 4 µg/kg. 

Quantification limits were calculated based on a signal/noise (S/N) ratio of 10. LOQs: OA 20 µg/kg; DTX-1 20 µg/kg; DTX-2 20 µg/kg; YTX 0.030 mg/kg; homoYTX 0.030 mg/kg; 45-OH YTX 0.030 mg/kg; 45-OH homoYTX 0.030 mg/kg; PTX-2 12 µg/kg; PTX-2-SA 12 µg/kg; putative 7-epi-PTX-2-SA 12 µg/kg; SPX-1 12 µg/kg; GYM 12 µg/kg. 

### 3.4. Alkaline hydrolysis

The hydrolysis procedure, as proposed by Mountfort *et al.* [54] with slight modifications, was carried out by adding 2.5 M NaOH (100% aqueous, 125 µL) to 1 mL of sample crude extract. The mixture was kept at 76 °C for 40 min. After cooling to room temperature, the extract was acidified with 2.5 M HCl (125 µL). The pH values of each extract after hydrolysis and addition of HCl ranged between 5.0 and 7.0, depending on the matrix.

### 3.5. Phytoplankton analysis

An aliquot of seawater (250 mL) was preserved in glutaraldehyde solution (final concentration 0.5%). *Dinophysis* spp. cells as well as other phytoplankton species were identified and counted using an inverted microscope (Olympus IX50) according to Utermöhl [55]. Toxic species were counted on the whole chamber’s bottom**.** Results were expressed as cells L^−1^. 

## 4. Conclusions

For the first time a complete analysis of the toxin profile of Croatian mussels has been carried out using the LC-MS/MS technique, which is characterized by high sensitivity and specificity. The obtained results showed OA as the main toxin contaminating Croatian mussels at that time. High concentrations of OA in mussels during the DSP toxic episode showed that a positive mouse bioassay could be fully attributed to the OA. Furthermore, the presence of only OA at concentrations which could endanger human health suggests *D*. *fortii* as the main organism responsible for the toxic event that occurred in Lim Bay. The presence of gymnodimine and spirolides in Croatian mussel has been detected for the first time while the presence of yessotoxin and pectenotoxin-2 is confirmed.
